# Author Correction: The CaMKII/NMDA receptor complex controls hippocampal synaptic transmission by kinase-dependent and independent mechanisms

**DOI:** 10.1038/s41467-018-07637-5

**Published:** 2018-12-03

**Authors:** Salvatore Incontro, Javier Díaz-Alonso, Jillian Iafrati, Marta Vieira, Cedric S. Asensio, Vikaas S. Sohal, Katherine W. Roche, Kevin J. Bender, Roger A. Nicoll

**Affiliations:** 1 Department of Cellular and Molecular Pharmacology, University of California, San Francisco, San Francisco, CA 94158 USA; 2 Department of Psychiatry, University of California, San Francisco, San Francisco, CA 94158 USA; 3 Receptor Biology Section, National Institute of Neurological Disorders and Stroke, National Institutes of Health, Bethesda, MD 20892 USA; 4 Department of Biological Sciences, University of Denver, Denver, CO 80208 USA; 5 Department of Neurology, University of California, San Francisco, San Francisco, CA 94158 USA; 6 Department of Physiology, University of California, San Francisco, San Francisco, CA 94158 USA

Correction to: *Nature Communications*; 10.1038/s41467-018-04439-7; published online 25 May 2018

The originally published version of this article contained errors in Fig. 5, for which we apologise. In panel c, the scatter graph was inadvertently replaced with a scatter graph comprising a subset of data points from panel d. Furthermore, the legends to Fig. 5c and d were inverted. These errors have now been corrected in both the PDF and HTML versions of the article, and the incorrect version of Fig. 5c is presented in the Author Correction associated with this Article.

**Fig. 1:**
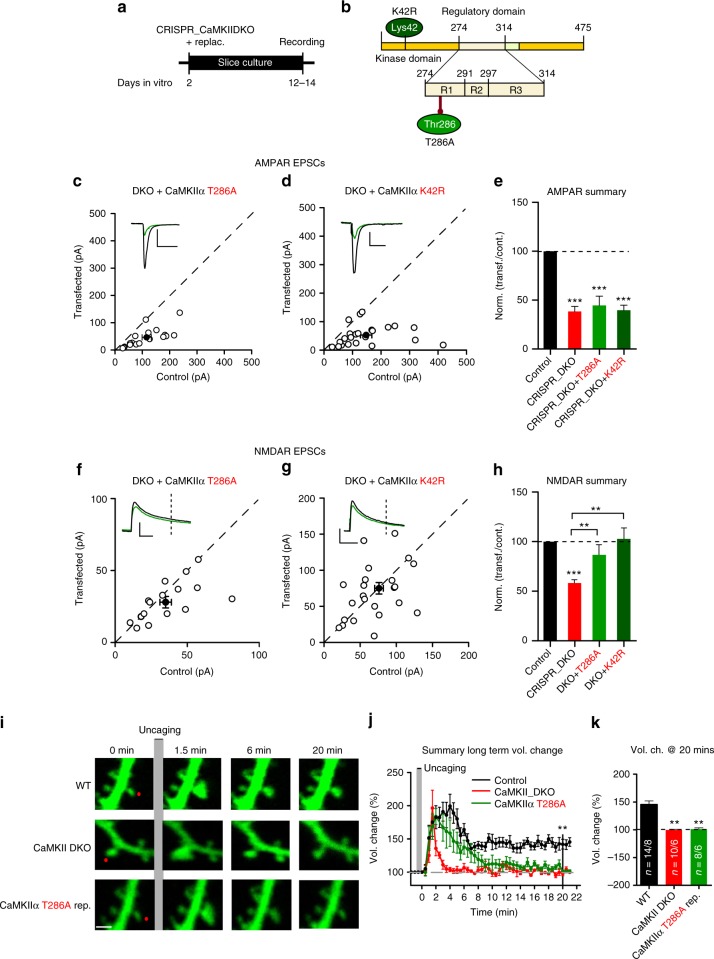
■

