# Supplementary material for: The CaMKII/NMDA receptor complex controls hippocampal synaptic transmission by kinase-dependent and independent mechanisms
Source: Nat Commun. 2018 May 25;9:2069. doi: 10.1038/s41467-018-04439-7 (PMC5970233; doi:10.1038/s41467-018-04439-7)
Supplement: Supplementary file 1 — Supplementary Information [file 41467_2018_4439_MOESM1_ESM.pdf]

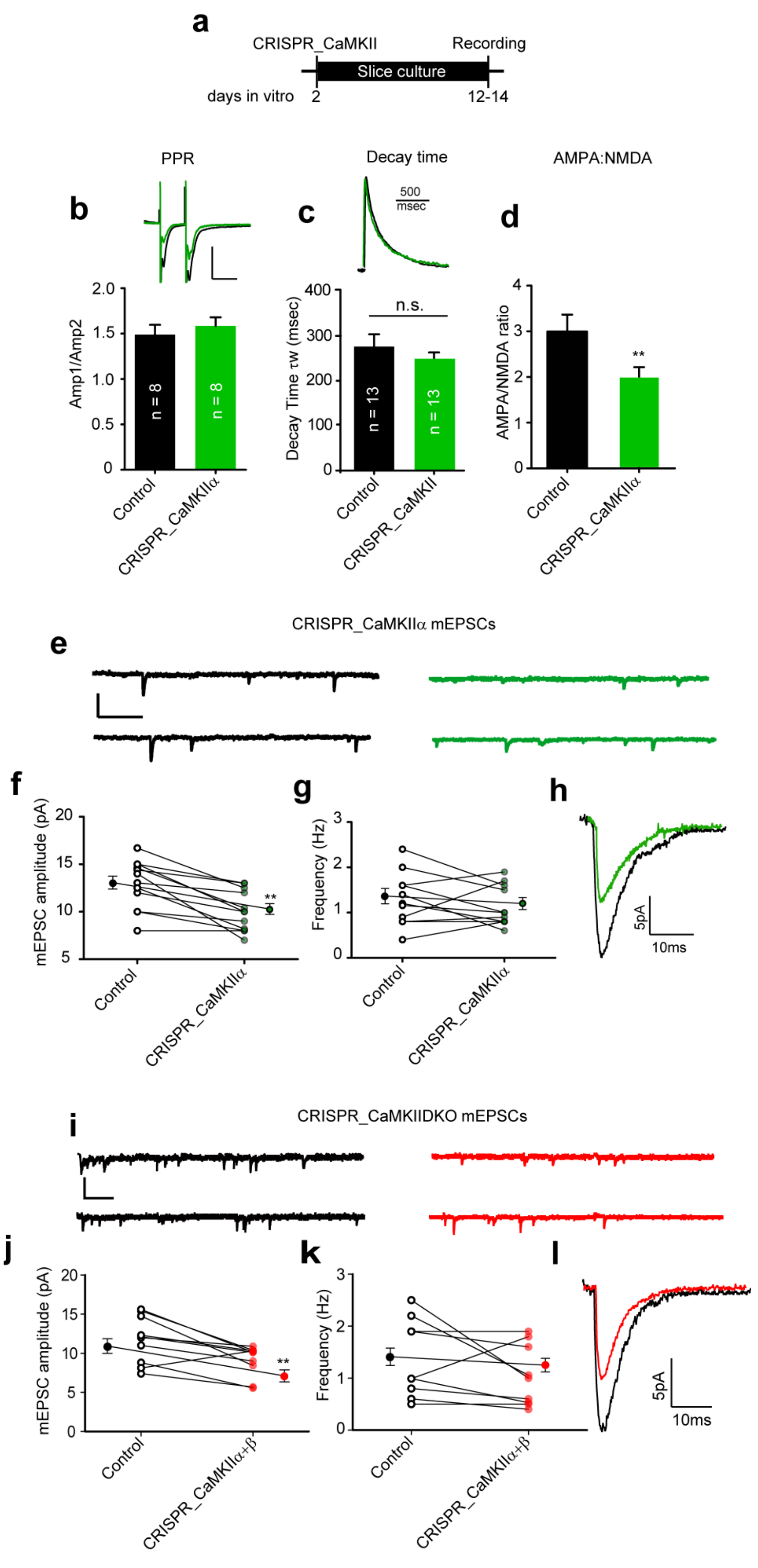

a
CRISPR_CaMKII
Recording
Slice culture
days in vitro 2
12-14
PPR
Decay time
AMPA:NMDA
b
c
d
500 msec
Amp1/Amp2
n = 8
n = 8
Control
CRISPR_CaMKIIα
n.s.
Decay Time τw (msec)
n = 13
n = 13
CRISPR_CaMKII
**
AMPA/NMDA ratio
CRISPR_CaMKIIα mEPSCs
e
f
g
h
mEPSC amplitude (pA)
Frequency (Hz)
5pA
10ms
CRISPR_CaMKIIDKO mEPSCs
i
j
k
l
CRISPR_CaMKIIα+β

Supplementary Figure 1 CaMKII elimination effects on pre- and postsynaptic electrophysiological parameters. **a** Timeline of the experiment. **b** Paired pulse ratio traces and bar graph for control and transfected cells, for CRISPR_CaMKIIα shown in figure 1. Mean values of AMPA second to first amplitude are 1.5 ± 0.1 n = 8 and 1.5 ± 0.1 n = 8, respectively. **c** Bar graph shows the decay time constant of NMDAR EPSCs recorded in NBQX at +40 mV (Control, 267.3 ± 12.3 (ms); CRISPR_CaMKII, 243.7 ± 7.9 (ms) p = 0.11). Scale bar, 0.5 s. This graph includes data from CRISPR_CaMKIIα and CRISPR_CaMKIIDKO. **d** Mean values of AMPA/NMDA ratio (Control, 3.1 ± 0.3; CRISPR_CaMKIIα, 2.2 ± 0.2, $p < 0.05$ unpaired t-test). **e** Sample traces of mEPSCs shown at a low gain and sweep speed. Control averaged trace (black) has been superimposed on the trace from a CRISPR_CaMKIIα cell (green). Scale bar, 10 pA, 500 ms. **f** Paired average mEPSCs amplitude of single pairs from control and transfected CRISPR_CaMKIIα cells. Mean ± SEM for control and transfected neurons are 12.9 ± 0.7 and 10 ± 0.6 respectively, **p = 0.003. Wilcoxon signed rank test. **g** Paired average mEPSCs frequency of single pairs from control and transfected CRISPR_CaMKIIα cells. Mean ± SEM for control and transfected neurons are 1.3 ± 0.2 and 1.1 ± 0.1, respectively, p = 0.06. **h** Sample traces of averaged mEPSCs at a high gain and sweep speed. Scale bar 5 pA and 10 ms. **i** Sample traces of mEPSCs shown at a low gain and sweep speed. Control trace (black) has been superimposed on the trace from a CRISPR_DKO cell (red). Scale bar, 10 pA, 500 ms. **j** Paired average mEPSCs amplitude of single pairs from control and transfected CRISPR_DKO cells. Mean ± SEM for control and transfected neurons are 11.7 ± 0.9 and 9 ± 0.6 respectively, **p = 0.003. **k** Paired average mEPSCs frequency of single pairs from control and transfected CRISPR_DKO cells. Mean ± SEM for control and transfected neurons are 1.3 ± 0.2 and 1 ± 0.2 respectively, p = 0.31. **l** Sample traces of averaged mEPSCs at a high gain and sweep speed. Scale bar 5 pA and 10 ms.

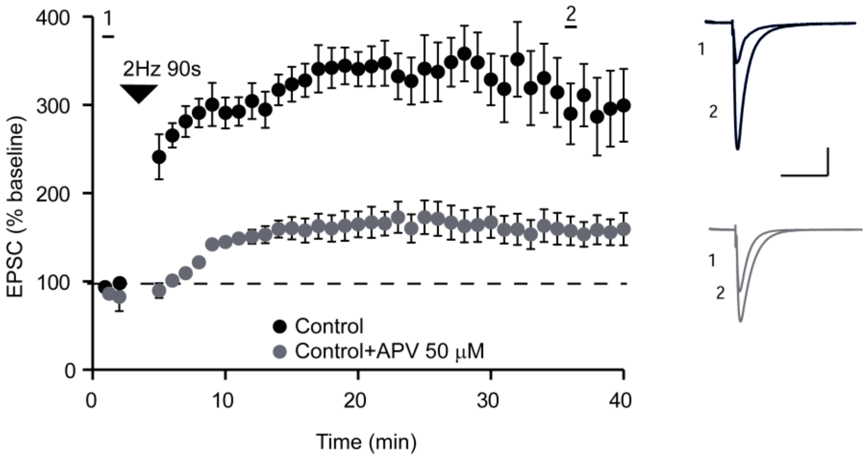

Supplementary Figure 2 Baseline run up in the presence of APV. Plots showing mean ± SEM AMPAR EPSC amplitude of control (black) and control in APV 50 µM (grey) pyramidal neurons normalized to the mean AMPAR EPSC amplitude before LTP induction (arrow). The control and APV experiments were interleaved. (Control, n=6; APV n = 8, p = 0.01 at 35 min). Mann-Whitney test was used to compare LTP at 35 min. Scale bars: 50 pA, 50 ms.

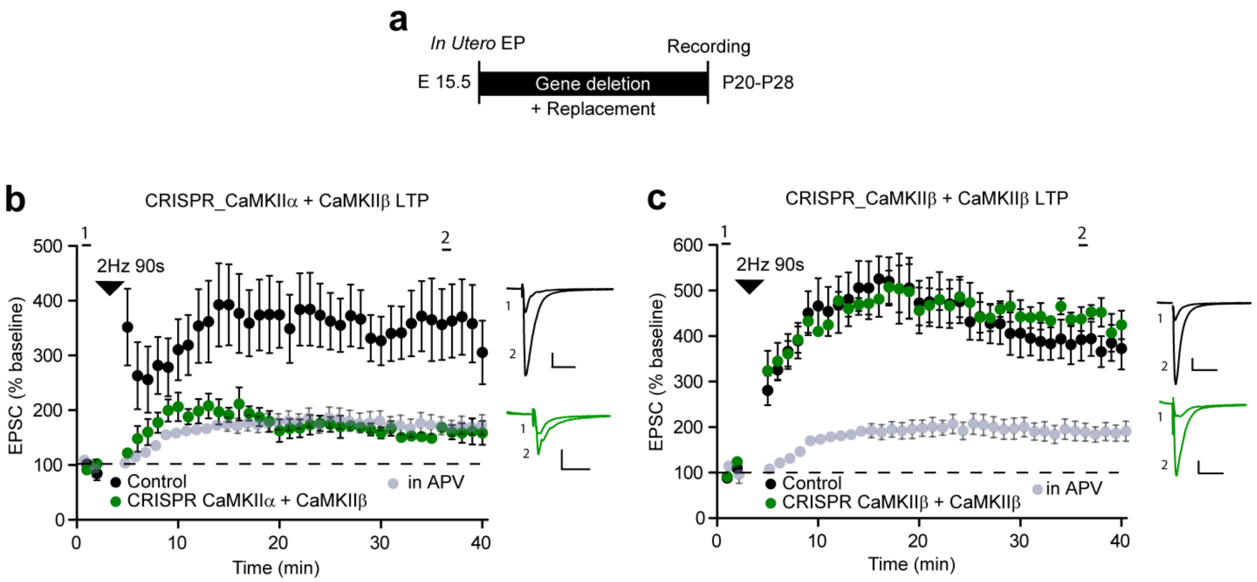

Supplementary Figure 3 Molecular replacement of CaMKIIβ confirms the key role of CaMKIIα in LTP. **a** Timeline of the *in utero* electroporation experiment. **b** CaMKIIβ itself is not able to rescue LTP after CaMKIIα deletion. Plots show mean ± SEM AMPAR EPSC amplitude of control (black) and transfected (green) CRISPR_ CaMKIIα + CaMKIIβ pyramidal neurons normalized to the mean AMPAR EPSC amplitude before LTP induction (arrow). (Control, n = 7; CRISPR_ CaMKIIα + CaMKIIβ n = 4, p = 0.006 at 35 min). **c** *In utero* electroporated neurons of CRISPR_CaMKIIβ + CaMKIIβ show normal LTP. Plots show mean ± SEM AMPAR EPSC amplitude of control (black) and transfected (green) CRISPR_CaMKIIβ + CaMKIIβ pyramidal neurons normalized to the mean AMPAR EPSC amplitude before LTP induction (arrow). (Control, n = 5; CRISPR_CaMKIIβ + CaMKIIβ n = 5, p = 0.34 at 35 min). Grey plots represent mean ± SEM AMPAR EPSC amplitude of LTP induction in APV 50 μM. Sample AMPAR EPSC current traces from control (black) and electroporated neurons (green) before and after LTP are shown superimposed to the right of each graph. Mann-Whitney test was used to compare LTP at 35 minutes (p values indicated above). Scale bars: 50 ms, 50 pA.

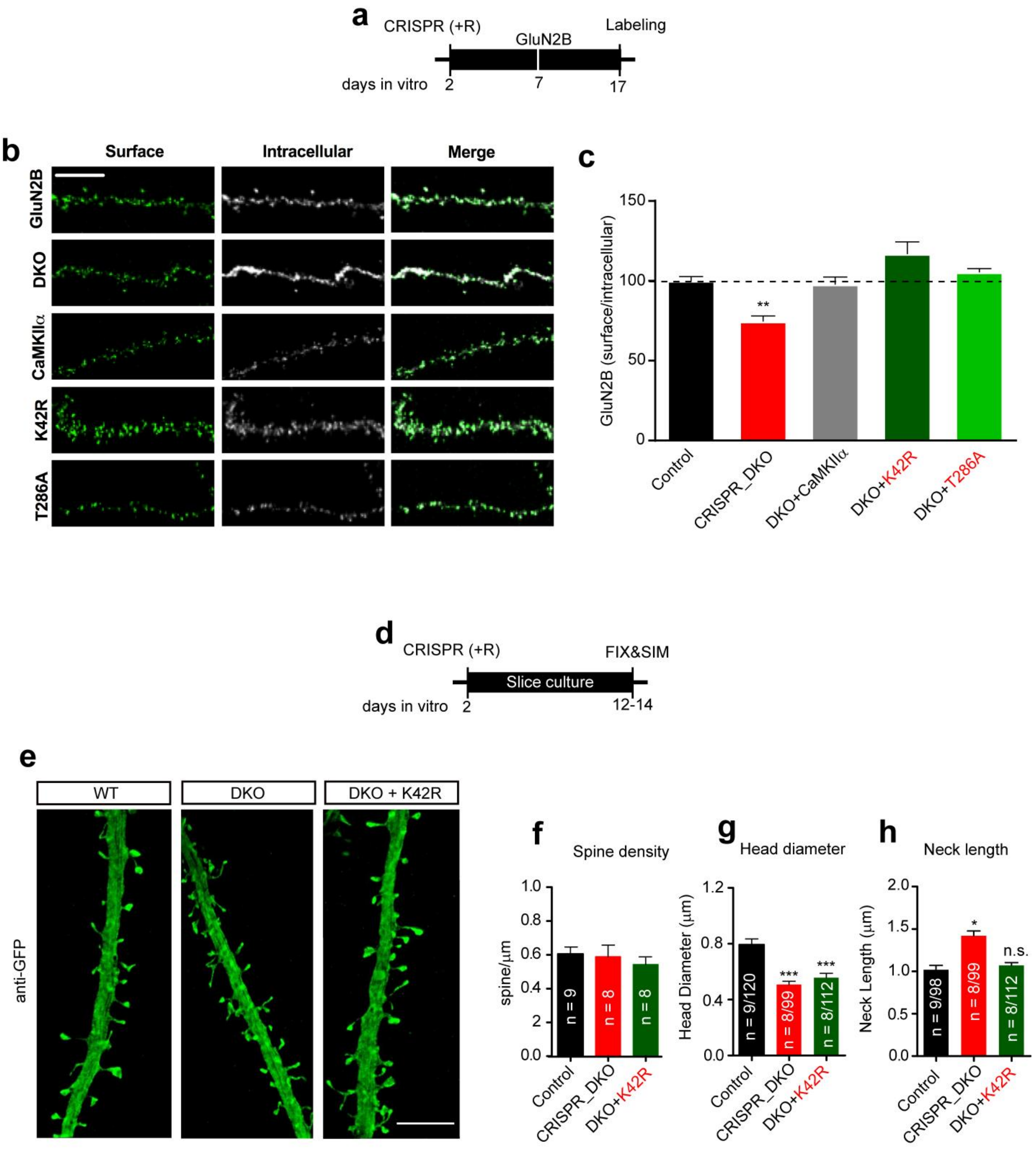

Supplementary Figure 4 GluN2B immunolabeling and spine morphology. **a** Timeline of the experiment. Hippocampal neurons were transduced with virus to knock down both CaMKII α and β isoforms. **b** Representative images of surface (in green) and intracellular (in grey)

expression of GFP-GluN2B. Scale bar 10 µm. **c** Quantitative analysis of (**b**). (**$p<0.001$; ***$p<0.0001$, n = 5). **d** Timeline of the experiment. **e** Sample images of primary apical dendrite from neurons expressing GFP, CRISPR_DKO GFP and DKO GFP + CaMKIIα K42R imaged using super-resolution structured illumination microscopy (SIM). For all morphological analysis, n = 7 neurons, 100 control spines; n = 8 neurons, 99 DKO spines, n = 8 neurons, 112 DKO + CaMKIIα K42R spines. Scale bar 5 µm. **f** Spine density on primary apical dendrites is unchanged following CaMKII DKO and in DKO + CaMKIIα K42R replacement (mean ± SEM number, GFP = 0.604 ± 0.038 µm; DKO = 0.59 ± 0.063 µm; DKO + CaMKIIα K42R = 0.543 ± 0.037 µm, $p>0.05$). **g** Spine head diameter is reduced by CaMKII DKO and not rescued by K42R replacement (GFP, diameter = 0.99 ± 0.05 µm; DKO, diameter = 0.63 ± 0.03 µm; DKO + CaMKIIα K42R, diameter = 0.69± 0.038 µm). **h** Spine neck length is reduced by CaMKII DKO but rescued by CaMKIIα K42R replacement (GFP, mean ± SEM number length = 1.019 ± 0.051 µm; DKO, mean ± SEM number length = 1.42 ± 0.056 µm; DKO + CaMKIIα K42R, mean ± SEM number length = 1.07 ± 0.03 µm). Scale bar 5 µm. Normalized data were analyzed using a one-way ANOVA. P values were calculated with the Mann-Whitney test (*$p < 0.01$; **$p < 0.001$; ***$p < 0.0001$).

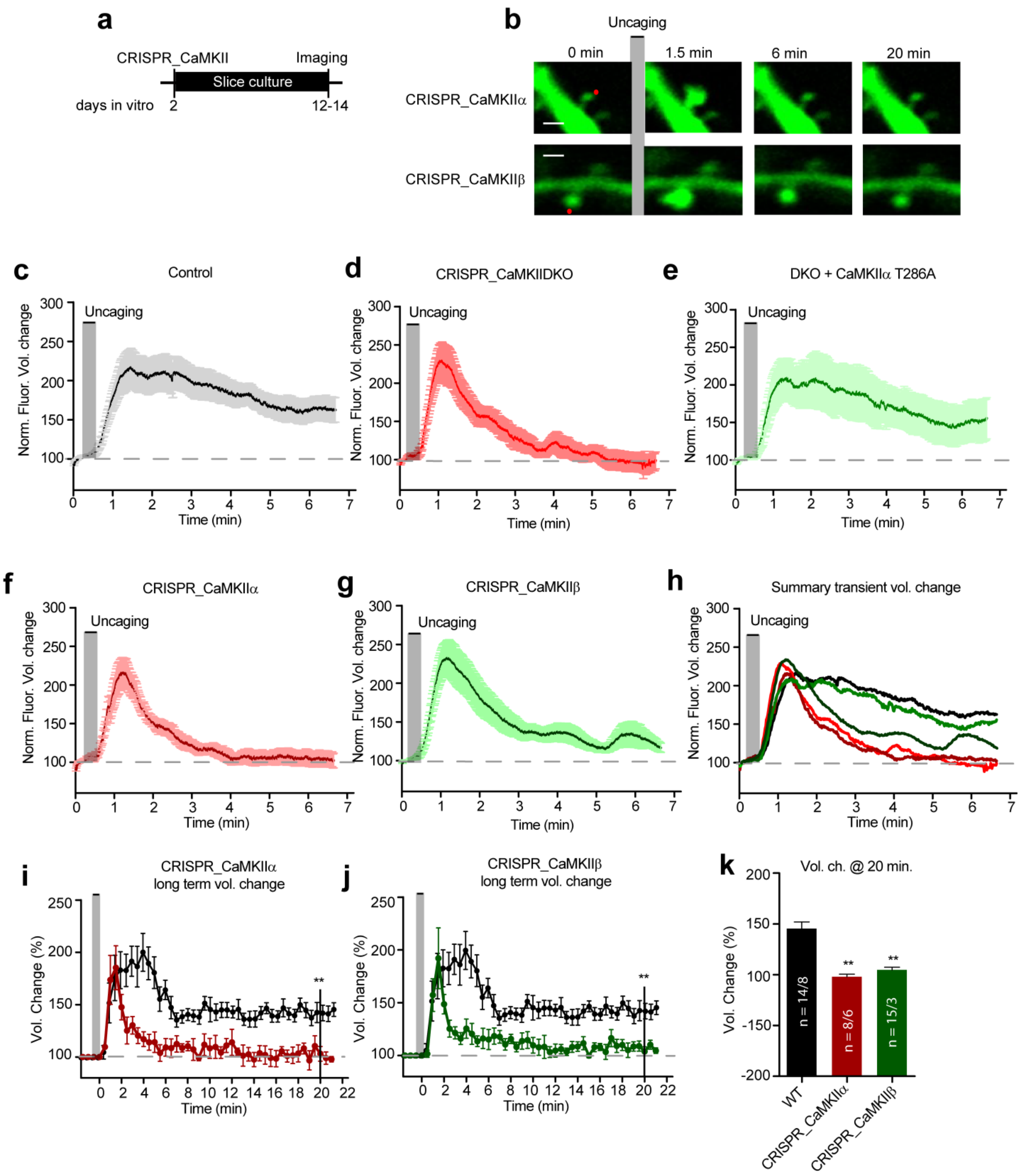

Supplementary Figure 5 Transient phase of structural LTP is independent from CaMKII. CaMKII is necessary to maintain long term volume change. **a** Timeline of the experiment. **b** Fluorescence GFP sample images of spine structural plasticity during sLTP in CRISPR_CaMKIIα and CRISPR_CaMKIIβ neurons. The red point indicates the spot of

glutamate uncaging. Scale bar 1 μm. **c - e** graphs are represented by the samples shown in figure 4. **c** Averaged fluorescence intensity normalized to baseline (F/F_0) for WT neurons transfected with FUGW GFP. Number of samples (spines/neurons) is 14/8; Black line represents mean ± SEM. **d** Averaged fluorescence intensity normalized to baseline (F/F_0) for CaMKII DKO neurons transfected with FUGW double gRNA GFP and Cas9. Number of samples (spines/neurons) is 10/6; Red line represents mean ± SEM. **e** Averaged fluorescence intensity normalized to baseline (F/F_0) for CaMKII DKO + CaMKIIα T286A neurons transfected with FUGW double gRNA GFP, Cas9 and pCAGGS CaMKIIα T286A. Number of samples (spines/neurons) is 8/6; Green line represents mean ± SEM. **f** Averaged fluorescence intensity normalized to baseline (F/F_0) for CaMKIIα KO neurons. Number of samples (spines/neurons) is 8/6; purple line represents mean ± SEM. **g** Averaged fluorescence intensity normalized to baseline (F/F_0) for CaMKIIβ KO neurons. Number of samples (spines/neurons) is 15/3; dark green line represents mean ± SEM. **h** Summary of the results from **c - g**. **i, j** Long term spine volume change of CRISPR_ CaMKIIα and CRISPR_ CaMKIIβ. Each point represents the mean ± SEM % of volume change every 30 seconds. Both graphs include control long term volume change data. (**k**) Bar graph of averaged volume change at 20 min. Values represent mean ± SEM as % of baseline volume. (Control = 145.5 ± 6; CRISPR_CaMKIIα = 100 ± 2.6; CRISPR_CaMKIIβ = 100.4 ± 6.5). **$p < 0.001$ (ANOVA test followed by Mann-Whitney test to calculate p value).

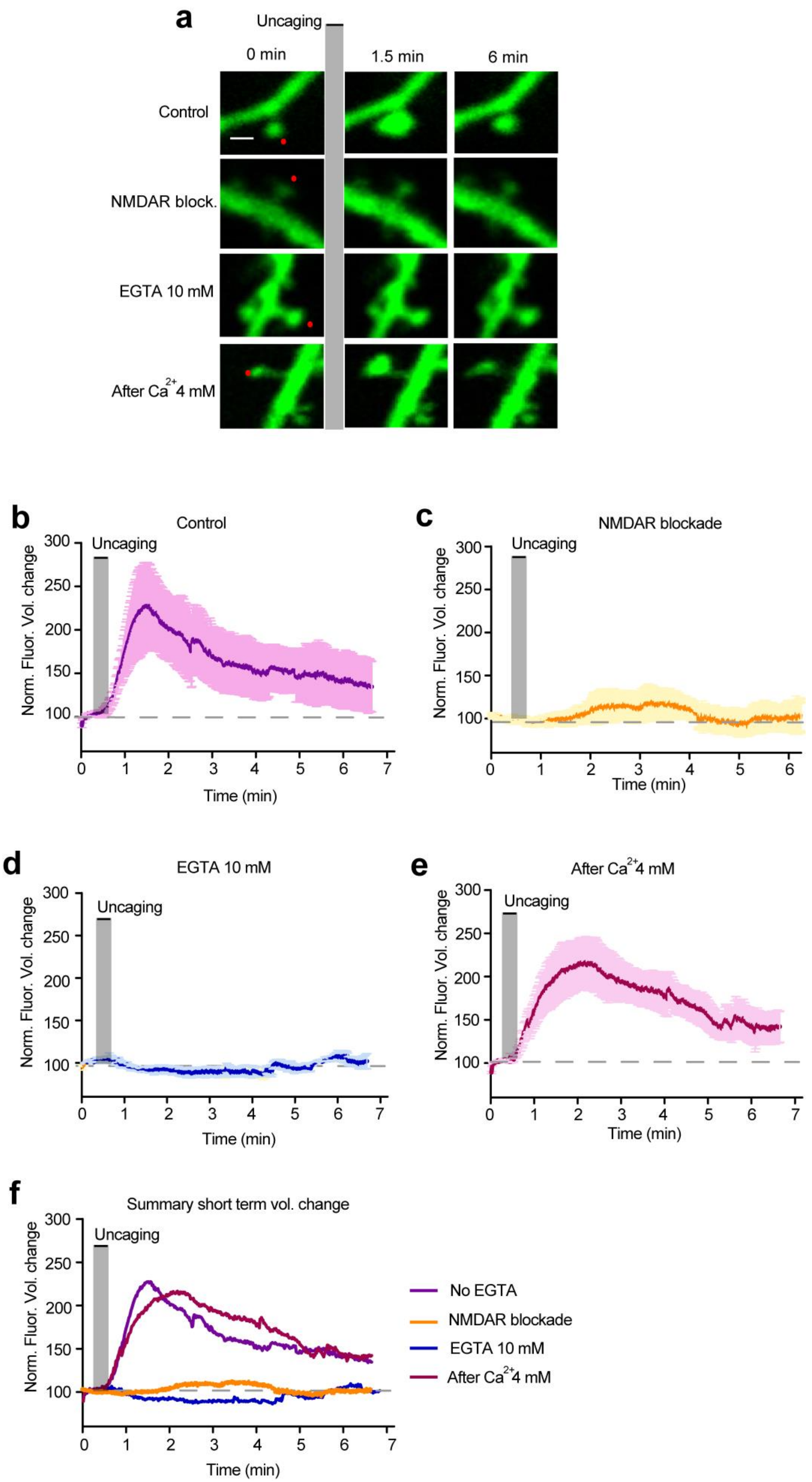

a
Uncaging
0 min
1.5 min
6 min
Control
NMDAR block.
EGTA 10 mM
After Ca2+ 4 mM
b
Control
c
NMDAR blockade
d
EGTA 10 mM
e
After Ca2+ 4 mM
f
Summary short term vol. change
Norm. Fluor. Vol. change
Time (min)
No EGTA
NMDAR blockade
EGTA 10 mM
After Ca2+ 4 mM

Supplementary Figure 6 Ca^{2+} influx through NMDAR fully accounts for the transient phase of sLTP. **a** Fluorescence GFP sample images of spine structural plasticity during sLTP. The red point indicates the spot of glutamate uncaging. Scale bar 1 μm. **b** Averaged fluorescence intensity normalized to baseline (F/F_0) for WT neurons transfected with FUGW GFP before adding EGTA. Number of samples (spines/neurons) is 8/4; line represents mean ± SEM. **c** Averaged fluorescence intensity normalized to baseline (F/F_0) for WT neurons transfected with FUGW GFP after adding CPP 30μM or APV 50μM in the ACSF. sLTP is fully prevented. Number of samples (spines/neurons) is 8/4; line represents mean ± SEM. **d** Averaged fluorescence intensity normalized to baseline (F/F_0) for WT neurons transfected with FUGW GFP after adding EGTA (10 mM) in the ACSF. sLTP is fully prevented. Number of samples (spines/neurons) is 8/4; line represents mean ± SEM. **e** Averaged fluorescence intensity normalized to baseline (F/F_0) for WT neurons transfected with FUGW GFP after adding EGTA (10 mM) in the ACSF and Ca^{2+} 4 mM. sLTP is fully rescued. Number of samples (spines/neurons) is 7/4; line represents mean ± SEM. **f** Summary of the results from **b** - **e**.

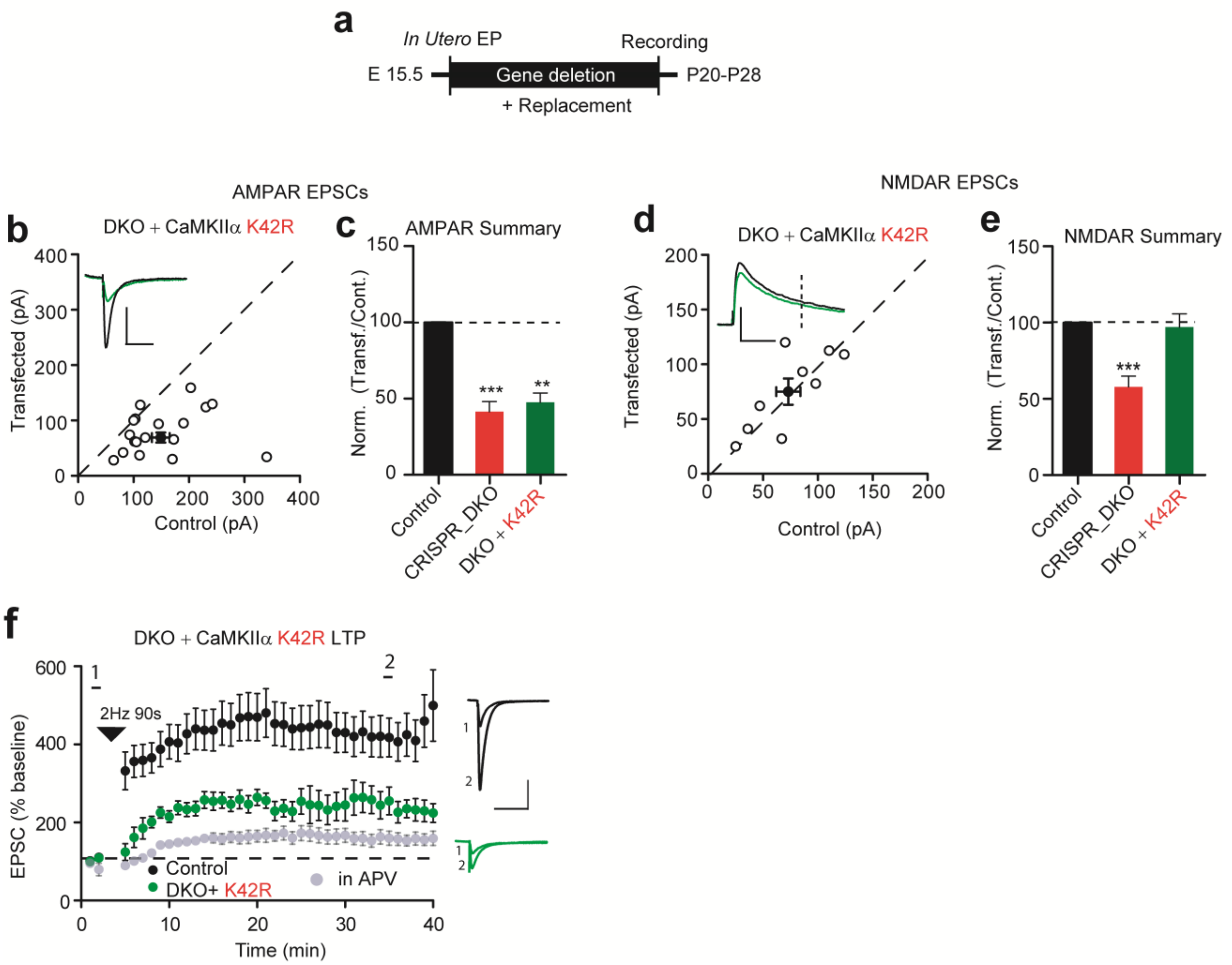

Supplementary Figure 7 Neurons expressing CaMIIα K42R shows residual LTP. **a** Timeline of the experiment. **b** Scatterplot showing amplitudes of AMPAR EPSCs for single pairs (open circles) of control and transfected cells of DKO + CaMKIIα K42R (n = 18 pairs). Filled circle indicates mean ± SEM. (Control = 149.1 ± 16.6; DKO + CaMKIIα K42R = 79.6 ± 9.2 p < 0.0001). (**c**) Bar graph of ratios normalized to control (%) summarizing the mean ± SEM of AMPAR EPSCs of values represented in **b** (59.1 ± 7.4 p = 0.001). DKO (red bar) from figure 1 is included in the graph. **d** Scatterplot showing amplitudes of NMDAR EPSCs for single pairs (open circles) of control and transfected cells of DKO + CaMKIIα K42R (n = 9 pairs). Filled circle indicates mean ± SEM. (Control = 73.7 ± 11.3; DKO + CaMKIIα K42R = 75.1 ± 12.1 p > 0.05). **e** Bar graph of ratios normalized to control (%) summarizing the mean ± SEM of NMDAR EPSCs of values represented in **d** (97 ± 8.8 p = 0.79). DKO (red bar) from figure 1 is included in the graph. **f** Plots showing mean ± SEM AMPAR EPSC amplitude of control (black) and

transfected DKO + CaMKIIα K42R (green) pyramidal neurons normalized to the mean AMPAR EPSC amplitude before LTP induction (arrow). (Control, n=14; DKO + CaMKIIα K42R n = 8, p = 0.009 at 35 min). Raw amplitude data from dual cell recordings were analyzed using Wilcoxon signed rank test (p values indicated above). Normalized data were analyzed using a one-way ANOVA followed by the Mann-Whitney test (**$p < 0.001$; ***$p < 0.0001$). Mann-Whitney test was also used to compare LTP at 35 min (p values indicated above). Scale bars: 50 ms, 50 pA.

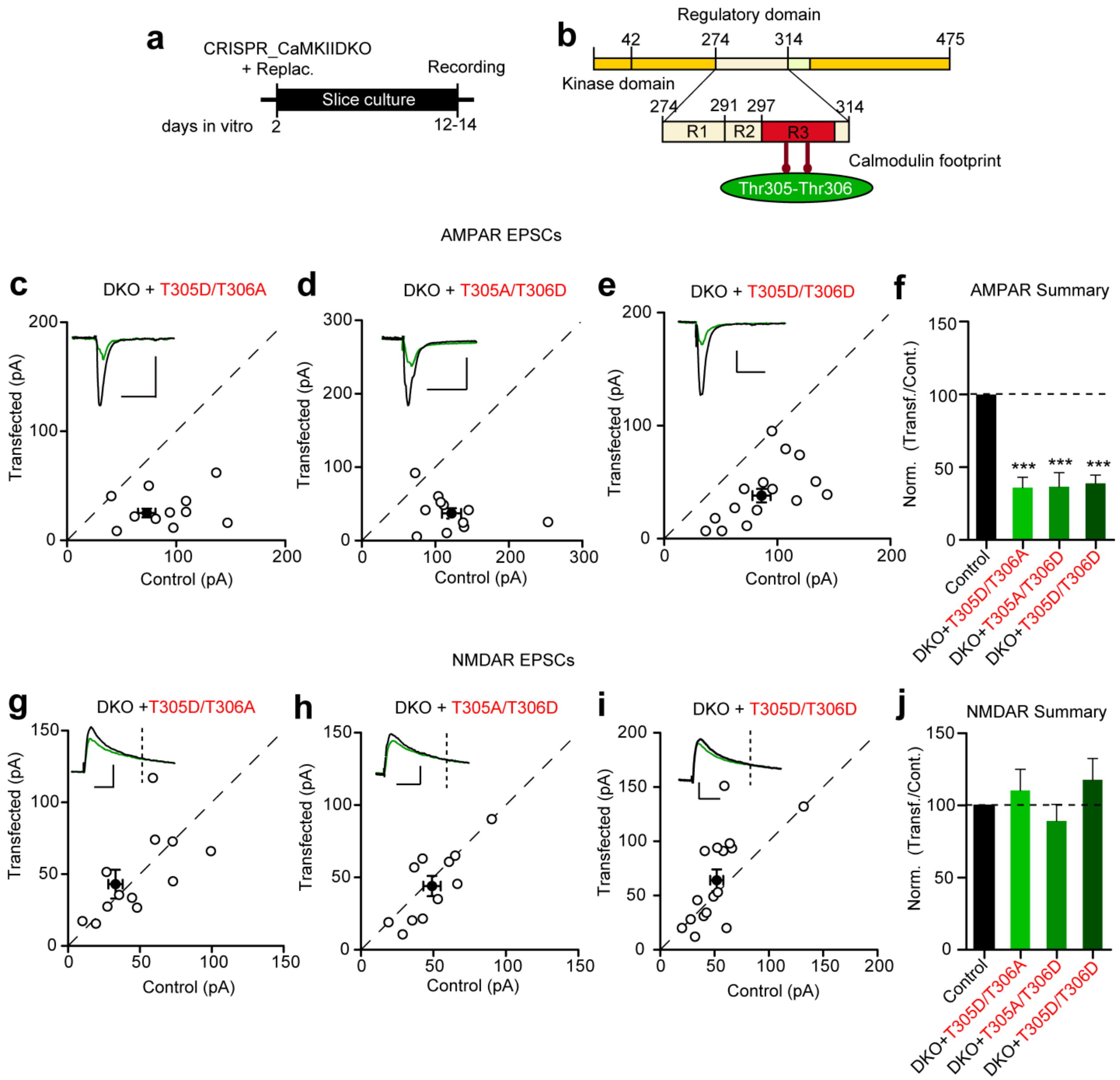

Supplementary Figure 8 The role of the calmodulin footprint inhibitory domain in the action of CaMKIIα. **a** Timeline of the experiment. **b** Scheme of the structural organization of CaMKIIα showing point mutations (T305/T306 residues). **c -e** Scatterplots showing amplitudes of AMPAR EPSCs for single pairs (open circles) of control and transfected cells of DKO + CaMKIIα T305D/T306A (**c**, n = 9 pairs), DKO + CaMKIIα T305A/T306D (**d**, n = 11 pairs) and DKO + CaMKIIα T305D/T306D (**e**, n = 16 pairs). Filled circles indicate mean amplitude ± SEM (**c**, Control = 73.4 ± 8; DKO + CaMKIIα T305D/T306A = 25.3 ± 4.6 $p < 0.004$; **d**, Control = 122.7 ± 13.7; DKO + CaMKIIα T305A/T306D = 37.8 ± 6.9 $p < 0.001$; **e**, Control = 87 ± 7.9; DKO +

CaMKIIα T305D/T306D = 38.8 ± 6.6 $p < 0.0001$). **f** Bar graph normalized to control (%) summarizing the mean ± SEM of AMPAR EPSCs of values represented in **c** (35.8 ± 7.2 $p < 0.0001$), **d** (36.5 ± 9.6 $p < 0.0001$) and **e** (38.9 ± 5.6 $p < 0.0001$). **g - i** Scatterplots showing amplitudes of NMDAR EPSCs for single pairs (open circles) of control and transfected cells of DKO + CaMKIIα T305D/T306A (**g**, n = 9 pairs), DKO + CaMKIIα T305A/T306D (**h**, n = 11 pairs) and DKO + CaMKIIα T305D/T306D (**i**, n = 16 pairs). Filled circles indicate mean amplitude ± SEM (**c**, Control = 36.8 ± 5.8; DKO + CaMKIIα T305D/T306A = 43.9 ± 10.9 $p > 0.05$; **d**, Control = 49.1 ± 6.1; DKO + CaMKIIα T305A/T306D = 44.7 ± 6.1 $p > 0.05$; **e**, Control = 52 ± 6.3; DKO + CaMKIIα T305D/T306D = 64.1 ± 10.5 $p > 0.05$). **j** Bar graph normalized to control (%) summarizing the mean ± SEM of AMPAR-eEPSCs of values represented in **c** (110.4 ± 14.6 $p = 0.60$), **d** (89.3 ± 11.4 $p = 0.73$) and **e** (117.7 ± 14.8 $p = 0.41$). Raw amplitude data from dual cell recordings were analyzed using Wilcoxon signed rank test (p values indicated above). Normalized data were analyzed using a one-way ANOVA followed by the Mann-Whitney test (***$p < 0.0001$). Scale bars: 50 ms, 50 pA.

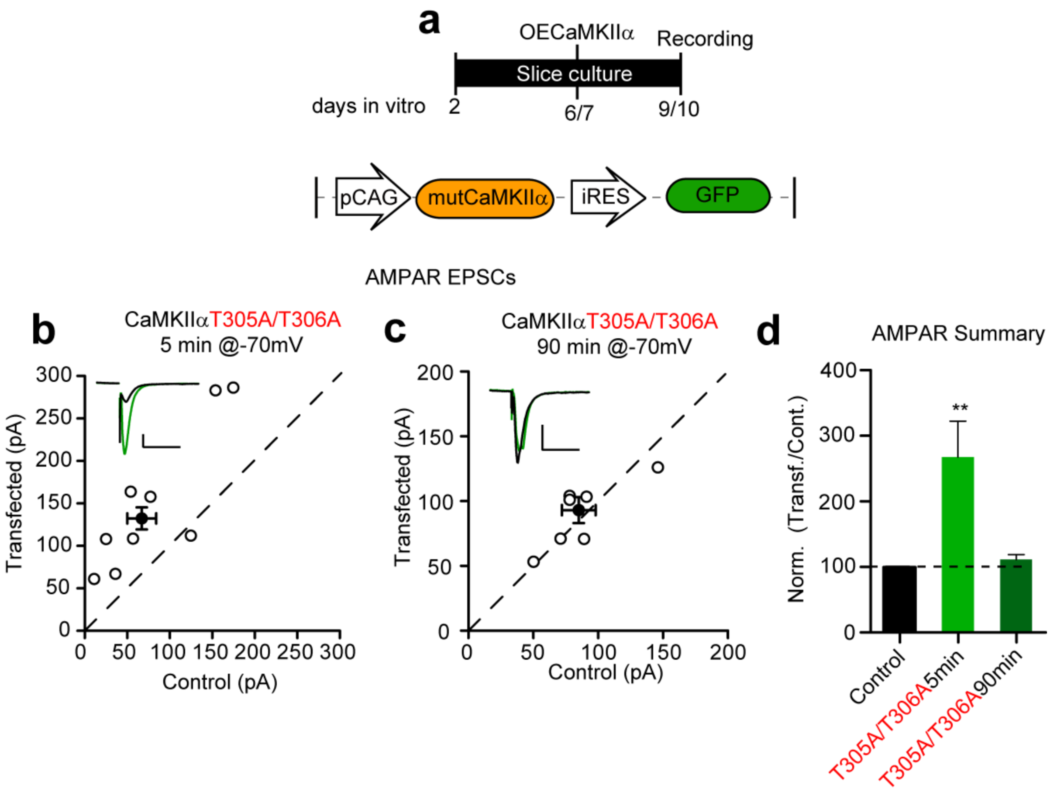

Supplementary Figure 9 Holding cells at -70mV for 90 minutes reverses AMPAR enhancement caused by the hypersensitive CaMKIIα overexpression. **a** Timeline of the experiment and map of the overexpressed plasmid. **b, c** Scatterplots showing amplitudes of AMPAR EPSCs for single pairs (open circles) of control and transfected cells of CaMKIIα T305A/T306A after 5 min at -70 mV (**b**, n = 9 pairs) and CaMKIIα T305A/T306A after 90 min at -70 mV (**c**, n = 7 pairs). Filled circle indicate mean amplitude ± SEM (**b**, Control = 82 ± 19 CaMKIIα T305A/T306A (5 min) = 132.68 ± 25.1 p < 0.001; **c**, Control = 85 ± 13; CaMKIIα T305A/T306A (90 min) = 93 ± 10 p > 0.05). **d** Bar graph normalized to control (%) summarizing the mean ± SEM of AMPAR-eEPSCs of values represented in **b** (268.8 ± 54.1 p = 0.009) and **c** (110.1 ± 9.8 p = 0.22). Raw amplitude data from dual cell recordings were analyzed using Wilcoxon signed rank test (p values indicated above). Normalized data were analyzed using a one-way ANOVA followed by the Mann-Whitney test (**p < 0.001). Scale bars: 50 ms, 50 pA.

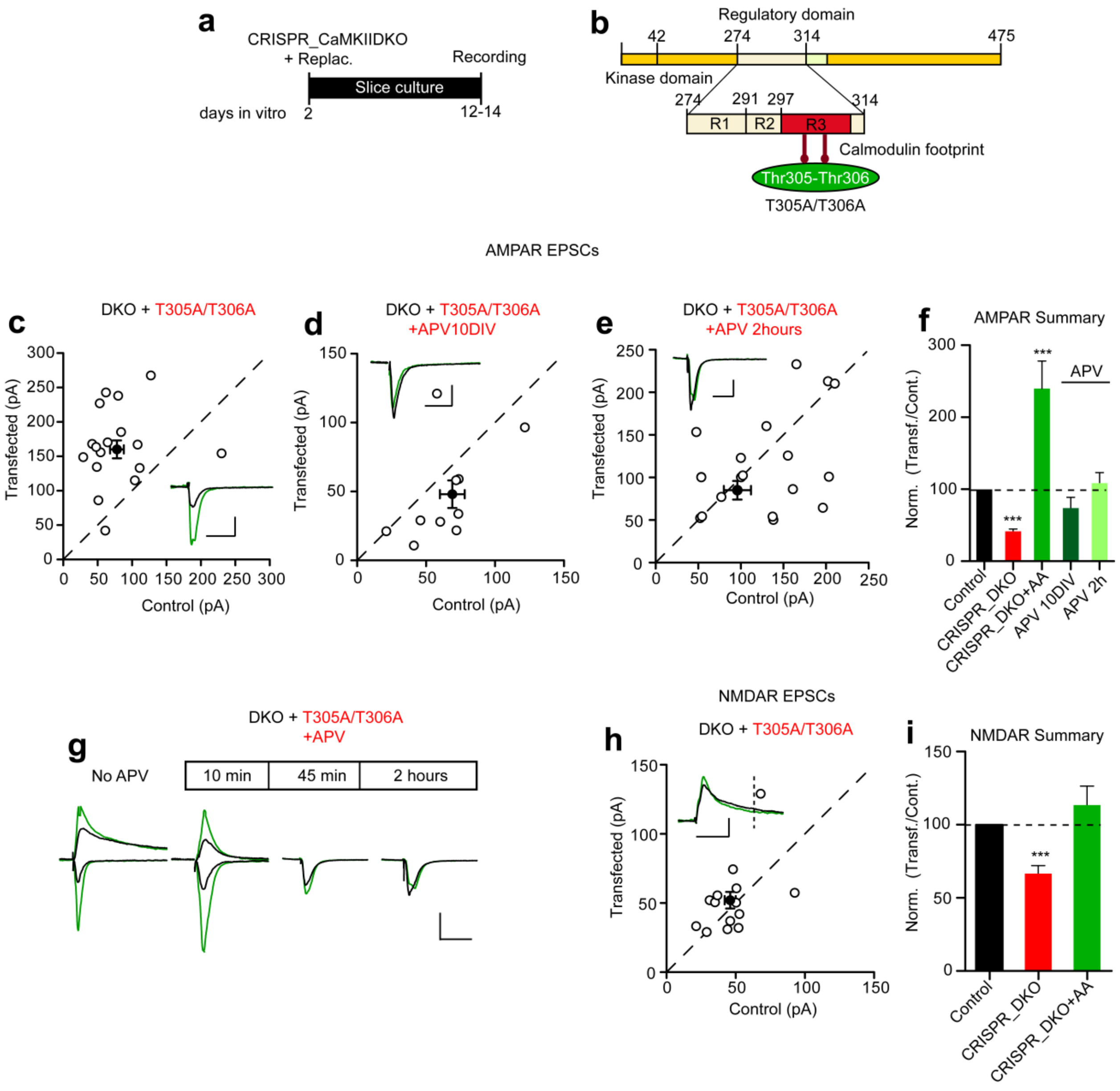

Supplementary Figure 10 Expression of the hypersensitive CaMKIIα enhances AMPAR EPSCs in an NMDAR - dependent manner. **a** Timeline of the experiment. **b** Scheme of the structural organization of CaMKIIα showing point mutations (T305/T306 residues). **c** - **e** Scatterplots showing amplitudes of AMPAR EPSCs for single pairs (open circles) of control and transfected cells of DKO + CaMKIIα T305A/T306A (**c**, n = 18 pairs), DKO + CaMKIIα T305A/T306A in APV during 10 DIV (**d**, n = 11 pairs) and DKO + CaMKIIα T305A/T306A in APV during 2 hours (**e**, n = 10 pairs). Filled circles indicates mean amplitude ± SEM (**c**, Control

= 78.4 ± 11; DKO + CaMKIIα T305A/T306A = 160.9 ± 10.9 p = 0.0009; **d**, Control = 69.1 ± 48.8; DKO + CaMKIIα T305A/T306A + APV 10 DIV = 48.8 10.5 p = 0.06; **e**, Control = 96.5 ± 16.6; DKO + CaMKIIα T305A/T306A + APV 2h = 85.9 ± 11.6 p > 0.26). **f** Bar graph normalized to control (%) summarizing the mean ± SEM of AMPAR EPSCs of values represented in **c** (250.4 ± 3 p < 0.0001), **d** (76.7 ± 15.2 p = 0.056) and **e** (114 ± 13.6 p = 0.58). **g** Sample traces of EPSCs at +40 mV and -70 mV in control (black) and CaMKIIα T305A/T306A replacement (green) before and after APV treatment. **h** Scatterplot showing amplitudes of NMDAR EPSCs for single pairs (open circles) of control and transfected cells of DKO + CaMKIIα T305A/T306A (**h**, n = 14 pairs). Filled circle indicates mean ± SEM. (Control = 46.7 ± 7.8; DKO + CaMKIIα T305A/T306A = 52.3 ± 6.8 p = 0.42). **i** Bar graph normalized to control (%) summarizing the mean ± SEM of AMPAR EPSCs of values represented in **h** (113.4 ± 13 p = 0.97). DKO (red bar) from figure 1 are included in the graph. Raw amplitude data from dual cell recordings were analyzed using Wilcoxon signed rank test (p values indicated above). Normalized data were analyzed using a one-way ANOVA followed by the Mann-Whitney test (***p < 0.0001). Scale bars: 50 ms, 50 pA.

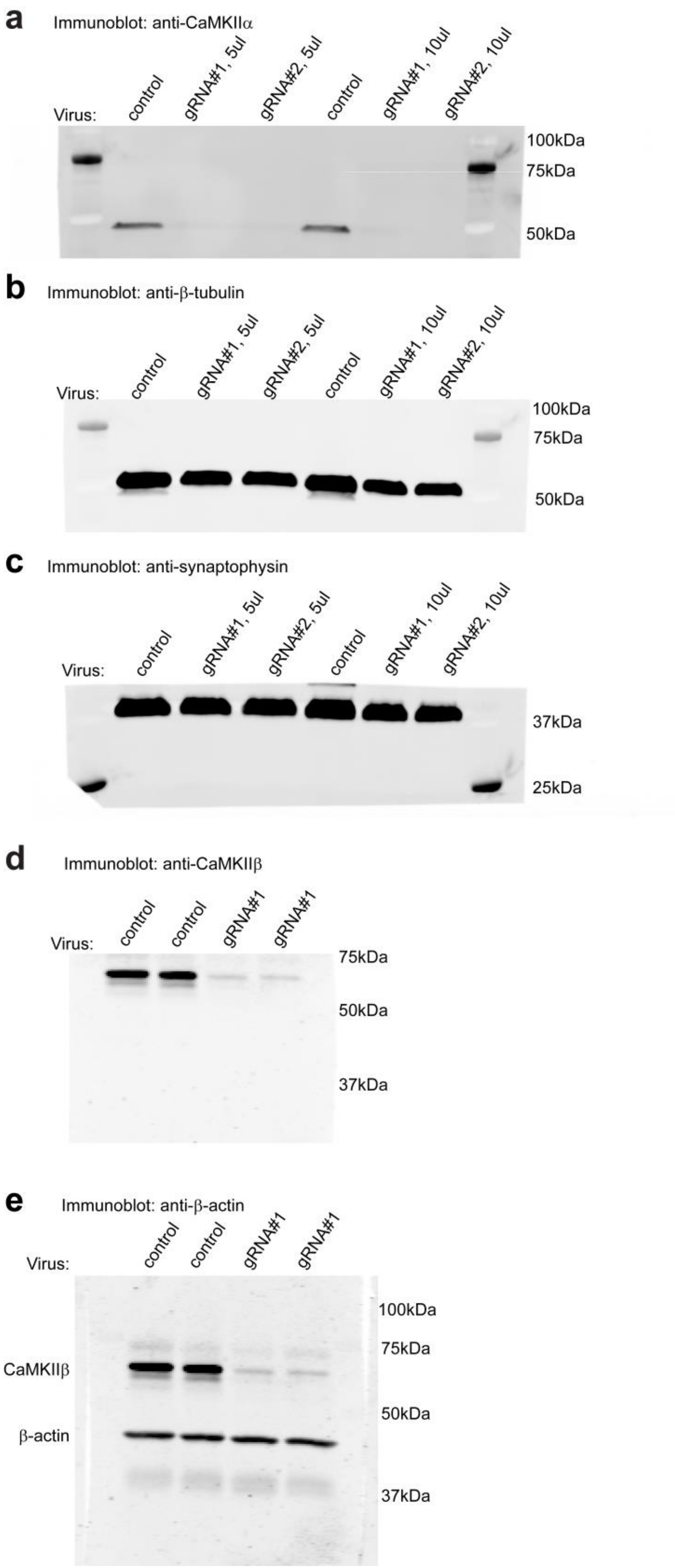

Supplementary Figure 11 Full size images of immunoblots shown in Figure 1. The name of the protein labeled and molecular weight markers are indicated.
